# The use of CorMatrix extracellular matrix for aortic root enlargement

**DOI:** 10.1186/s13019-014-0178-5

**Published:** 2014-11-19

**Authors:** Derek R Brinster, Jay A Patel

**Affiliations:** Department of Cardiothoracic Surgery, Virginia Commonwealth University, Richmond, VA USA; Divisions of Cardiothoracic and Vascular Surgery, Virginia Commonwealth University Medical Center Medical College of Virginia Campus, West Hospital Building, 7th Floor, South Wing, 1200 East Broad Street, Richmond, 23298-0068 VA USA

**Keywords:** Aorta, Aortic valve replacement, Aortic root enlargement, CorMatrix, Extracellular matrix

## Abstract

This manuscript presents a published record of the use and efficacy of the extracellular matrix CorMatrix and why it may be superior to other materials used for aortic root enlargement. The potential benefits of an extracellular matrix are the natural ingrowth and development of native arterial cells and structure.

**Electronic supplementary material:**

The online version of this article (doi:10.1186/s13019-014-0178-5) contains supplementary material, which is available to authorized users.

## Background

Aortic root enlargement (ARE) is a surgical procedure used to widen the annulus of the aortic root to facilitate aortic valve replacement (AVR). ARE is sometimes necessary to prevent patient-prosthesis mismatch, which occurs when the area of the prosthesis is insufficient relative to the size of the patient, and is associated with higher rates mortality [1]. Both the orifice area of the prosthesis and the resulting pressure gradient across the aortic valve are important determinants to successful AVR. Procedures used to enlarge the aortic root include the Nicks’ Procedure which is the most common, Manougian Technique, Seybold-Epting Technique, modified Bentall Procedure, and aortoventriculoplasty. During AVR, an effective means towards repair and augmentation of the aortic root is necessary. Materials currently used for reconstruction are Dacron (cloth), polytetrafluroethlyene (polymer), and bovine/patient derived pericardium [2]. Significant drawbacks to these materials include difficulty in resorption and difficulty in promoting full functioning of the reconstructed area [2]. An important alternative to these methods is the CorMatrix ECM, an extracellular matrix (ECM) derived from porcine small intestinal submucosa [3], which includes a matrix of structural proteins normally found outside cells that provide tissue support. This manuscript will review the initial use of CorMatrix in ARE.

## Case presentation

The following seven patients had an ARE procedure performed between October 2011 and April 2013 due to small annular size during AVR:

*Case 1 patient.* A 79-year-old female who presented with significant shortness of breath, flash pulmonary edema, severe aortic stenosis, ventricular hypertrophy, and diastolic heart failure.

*Case 2 patient.* A 82-year-old male who presented with shortness of breath, stenosis of the coronary artery (LAD), severe atherosclerosis of the aortic valve, and a thin aortic root.

*Case 3 patient.* A 40-year-old female who presented with aortic valve murmur and worsening aortic stenosis of her BAV.

*Case 4 patient.* A 75-year-old male who presented with a history of heart failure, two surgeries involving a coronary artery bypass graft, aortic stenosis, COPD, hypertension, hyperlipidemia, atrial fibrillation, and who had a biventricular ICD.

*Case 5 patient.* A 61-year-old African-American female who presented with angina and dyspnea with exertion, aortic stenosis, and a history of coronary artery disease and stents.

*Case 6 patient.* A 44-year-old female who presented with a congenital abnormality in her aortic valve, which was accompanied with severe aortic stenosis.

*Case 7 patient.* A 76-year-old female who presented with nonischemic cardiomyopathy, lowered ejection fraction, and who had a biventricular ICD.

**Figure 1:**
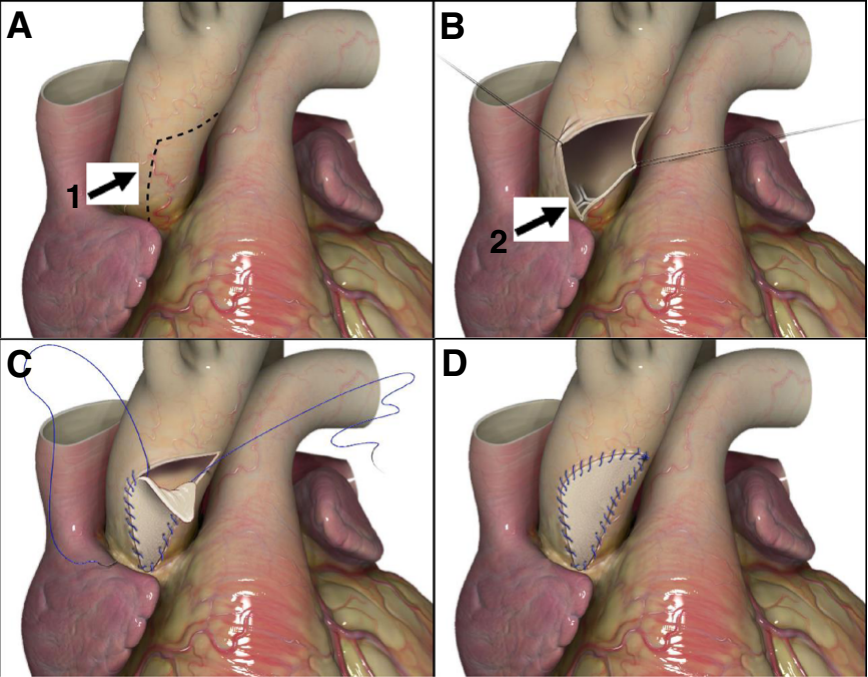
**ARE procedure. (A)** Incision in the aortic root (1). **(B)** Opening of the aortic root and exposure of the aortic valve (2). **(C)** Suturing of CorMatrix patch after AVR. **(D)** Sealed aortic root.

ARE was done by utilizing the Nick’s procedure to increase the size of the aortic valve implantation. The annular size was increased by extending the aortotomy down to the noncoronary sinus and traversing the annulus down to the anterior leaflet of the mitral valve (Figure 1A,B). The aortic valve prosthesis was then implanted into the enlarged annulus, the patch was then used to augment the aortic root to ensure no impingement on the bioprosthetic, and the defect in the annulus was closed with the CorMatrix ECM patch strut (Figure 1C,D).

All patients had successful aortic root enlargement. The average size of the bioprosthetic valve was 23.3 ± 1.4 mm, and the average mean prosthetic gradient was 12.5 mmHg ±4.3 mmHg, the average peak prosthetic gradient was 21.6 mmHg ±6.2 mmHg. There were no postoperative reoperations for bleeding, no strokes, and no operative mortality. Average length of stay was 10.3 days ±4.1 days. Routine postoperative transthoracic echocardiograms performed revealed normal aortic root appearance.

## Conclusions

There is no definitive conclusion on the best material to use for the reconstruction of cardiac tissue, but an exceptional material would possess tensile strength, hemostatic abilities, calcification resistance, and inability to evoke an immune response [4]. There is evidence that CorMatrix possesses most of these characteristics as it is able to withstand 1200+ mmHg of pressure, and has not been demonstrated to undergo calcification nor instigate an immune response [5]. According to the successful outcomes and rapid amelioration of the patients discussed, who displayed no apparent complications with regards to the implanted material, CorMatrix ECM may be better than alternative materials *in vivo*. The most significant drawbacks of the alternative materials include tearing, dislodgement, erosion, and calcification [4]. Bovine pericardium possesses additional problems including its cellularity which does not promote growth and stimulates an immune response [4]. Based on their compositions, Dacron and polytetrafluoroethylene are unable to promote growth of host tissue as well. CorMatrix ECM is a non-cellular material consisting of only structural proteins, which effectively mimics the natural environment of cells. CorMatrix has been found to readily undergo reabsorption [3] and promotes the growth of a new population of the host’s cells [4]. For example, studies of CorMatrix ECM on augmentation cystoplasty showed significant degeneration of the matrix at four weeks post operation, which was accompanied with the proliferation of host ECM and vascularization [3]. By 8 to 12 weeks after the surgery, the implanted matrix was undetectable [3]. A patient who underwent ARE with CorMatrix, displayed normal aortic morphology 30 weeks after the surgery [5]. CorMatrix offers the added benefits of a lower probability of infection and ease of implementation. Although the use of CorMatrix for ARE procedures is promising post-operatively, further studies involving a longer follow up period for the patients (i.e. echocardiography) could prove useful and allow for the assessment of longer term efficacy of CorMatrix ECM.

## Consent

Patients in this study have provided written consent to the use of their medical data for academic endeavors, such as submission of a case report to a medical journal, through a consent form that was provided to the patient and/or patient family prior to surgery. A copy of the written consent is available for review by the Editor-in-Chief of this journal.

## Authors’ contributions

DB and JP have both contributed to data acquisition, writing of the manuscript and making of the figures. Both authors read and approved the final manuscript.

## Authors’ original submitted files for images

Below are the links to the authors’ original submitted files for images. Authors’ original file for figure 1

## Abbreviations

ARE: Aortic root enlargement
AVR: Aortic valve replacement
ECM: Extracellular matrix

## References

[1] Losenno KL, Gelinas JJ, Johnson M, Chu MW (2013). Defining the efficacy of aortic root enlargement procedures: a comparative analysis of surgical techniques. Can J Cardiol.

[2] Sündermann SH, Biefer HR, Emmert MY, Falk V (2012). Use of extracellular matrix materials in patients with endocarditis. Thorac Cardiovasc Surg.

[3] Badylak SF, Kropp B, McPherson T, Liang H, Snyder PW (1998). Small intestinal submucosa: a rapidly resorbed bioscaffold for augmentation cystoplasty in a dog model. Tissue Eng.

[4] Quarti A, Nardone S, Colaneri M, Santoro G, Pozzi M (2011). Preliminary experience in the use of an extracellular matrix to repair congenital heart diseases. Interact Cardiovasc Thorac Surg.

[5] Gerdisch MW, Akinwande AO, Matheny RG (2010). Use of a novel acellular xenograft as a path for aortic annular enlargement during aortic valve replacement. Innovations (Phila).

