# Supplementary figures and images for: The use of CorMatrix extracellular matrix for aortic root enlargement
Source: J Cardiothorac Surg. 2014 Nov 19;9:178. doi: 10.1186/s13019-014-0178-5 (PMC4247869; doi:10.1186/s13019-014-0178-5)

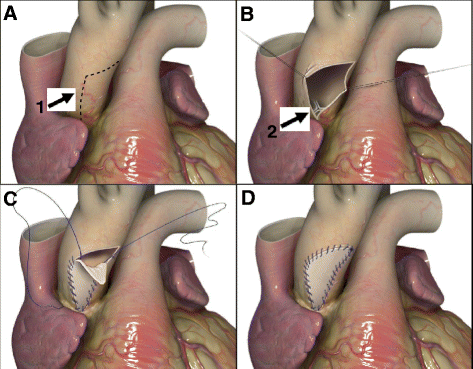

Supplement: Supplementary file 1 — Authors’ original file for figure 1 [file 13019_2014_178_MOESM1_ESM.gif]
